# 

**Published:** 1856-07

**Authors:** 


The Editor is anxious to give free scope to the expression of opinion, but does not hold himself responsible for the opinions of those of his contributors who attach their *names* to the articles written by them.

*The Nursing Scheme.* The Poor-Law Board have addressed a letter to the Poor-Law Inspectors, suggesting that in Workhouses, such female inmates as may be trustworthy, competent, and willing for the duties, be employed in the sick wards as assistants to the paid nurses, with a view of being taught the duties of a nurse in such a manner as to become nurses on their own independent account. Thus far Dr. Sieveking's admirable suggestion has gained ground.

*Food of Infancy.* Dr. Huxley, of Maidstone, referring to Dr. Brown's paper in last number, directs attention to the following quotation from Burns's Midwifery, on the Food of Infancy :

"The quantity of cream is greatest in ewes' milk, next in that of woman, the goat, the cow, and then the ass and the mare. The proportion of whey is greater in the milk of mares and women, than of the cow or the sheep. With regard to the caseous part, it is greatest in the milk of sheep, the goat, the cow, the ass, the mare, in the order in which they stand, *and it is little in that of woman.* Sugar, again, is most abundant in the milk of the mare and woman, and less so in that of the goat, the sheep, and the cow. *Woman's milk contains more cream than cow's milk;* yet no butter can be made from it. It contains much whey, and yet it scarcely ever becomes sour by exposure to air. * * * From these remarks, it follows that if a child be not suckled, the best food will be milk resembling that of women, and the nearest is asses' ; but as this cannot always be procured, we must change that of cows, so as to diminish the proportion of curd and increase that of sugar and cream, which is done by adding *an equal quantity of water, a sixth part of fresh cream, or less, if it be rich, and a little sugar.* * * * This is to be mixed just as it is required ; for, by standing, it acquires bad properties."

*Dr. Southwood Smith.* A proposal is on foot to present a fitting testimonial to the veteran Sanitarian, Dr. Southwood Smith. A great number of noblemen and members of the profession have joined in this undertaking, which we heartily recommend to all our readers. Few men deserve the recognition of their countrymen more than this distinguished and laborious sanitary reformer.

*Medical Officers of Health Association.* The Medical Officers of Health in the metropolis have formed themselves into a scientific association. Mr. Simon is elected President, and Dr. Hillier Secretary. Their meetings are held at the Board of Health Office, Whitehall. We anticipated this movement, and have already pointed out (see *Journal of Public Health* for December, 1855), the importance of such a society, scientifically and vigorously conducted.

*The Smoke Nuisance in Scotland.* Dr. John Webster writes that during his visit to Scotland in the autumn of last year, his attention was directed to the extraordinary prevalence of the smoke nuisance. Thus, for twenty miles, more or less, to the north, north-east, and south of Glasgow, and in that city itself, the whole atmosphere is loaded with a thick fog, caused by the smoke arising from the chimneys of factories, ironworks, &c. The same condition prevails at Aberdeen, Arbroath, Dundee, even in Edinburgh, and in other parts of Scotland; so that the proverbial smokiness of London, under its improved condition, sinks into insignificance in comparison with what is observed in the north.

*To Literary Contributors.* We regret that, owing to the length of Mr. Keith Johnston's valuable communication, we are obliged to hold over till next number, Part V of the Dictionary of Foods and Drinks, and communications by Dr. Winn, Mr. Houghton, Mr. Nicholas, and other contributors.

### BOOKS RECEIVED.

ACLAND (Henry W., M.D., F.R.S.) Memoir on the Cholera at Oxford in 1854. 4to. pp. 176. London: Churchill. 1856.

ALEXANDER (William, M.D.) On the Adulteration of Food and Drink. pp. 32. London: Longman and Co. 1856.

BARNES (Robert, M.D.) Preliminary Report of the Sanitary Condition of Shoreditch. London: 1855.

BLAIR (Daniel, M.D.) Report on the recent Yellow Fever Epidemic of British Guiana. pp. 91. London: Churchill. 1856.

BRINTON (William, M.D.) On the Medical Selection of Lives for Assurance. pp. 58. London: Churchill. 1856.

MACLOUGHLIN (David, M.D.) Did the General Board of Health make any Bedside Researches to ascertain the connexion which exists between Epidemic Diarrhœa and Epidemic Cholera? A Letter to John Simon, Esq., F.R.S. Pamphlet. London: T. Richards. 1856.

NOBLE (Daniel, M.D.) On some of the Vices of the Poor-Law Medical Relief System. Pamphlet. Manchester: Harrison. 1856.

PAINE (H. J.) Third Annual Report on the Sanitary Condition of Cardiff. Pamphlet. Cardiff: 1856.

PAVY (F. W., M.D.) On the Nature of the Normal Destruction of Sugar in the Animal System. Pamphlet. London: 1856.

ROBERTON (John). On Defects in the Plan of Construction and Ventilation in most of our Hospitals for the Reception of the Sick and Wounded. Manchester: 1856.

RUMSEY (H. W.) Essays on State Medicine. pp. 424. London: Churchill. 1856.

The Hospital System of London. pp. 53. London: Davy & Son. 1856.

A Brief Summary in Plain Language of the most important Laws concerning Women. Pamphlet. London: John Chapman. 1854.

The Education of the Imbecile, and the Improvement of Invalid Youth. Pamphlet. Published for behoof of the School for Invalid and Imbecile Children. Edinburgh: 1856.

On the Medicinal Properties of the Mineral Waters of Vichy. Pamphlet. Published for the Vichy Water Company. London: 1856.

---

### IN EXCHANGE.

Scottish Review.
Gazette Médicale de Paris.
Dublin Hospital Gazette.
Literary Gazette.
Glasgow Medical Journal.
Psychological Journal.
Association Medical Journal.
American Journal of the Medical Sciences.
New York Journal of Medicine.
Montreal Medical Chronicle.
Ranking and Radcliffe's Abstract of the Medical Sciences.
Chemist.
Veterinarian.
Charleston Medical Journal.
Edinburgh Medical Journal.
Asylum Journal of Mental Science.
Quarterly Journal of the Chemical Society.
Aerzliches Intelligenz-Blatt.

**To New Subscribers to the Journal of Public Health.**

The Publisher begs to intimate that new Subscribers wishing to have the series of the Journal complete from the first number, can obtain sets of Vol. I, at Subscriber's prices, 10s. post free, by application to him, including Post-Office Order, payable at the Charing Cross Branch to THOMAS RICHARDS.

THE JOURNAL OF PUBLIC HEALTH is sold by all Booksellers, and is equally suited for the general and professional reader, for the scientific society, and the private library.

LONDON: T. RICHARDS, 37, GREAT QUEEN STREET.

